# Generation and functional characterization of a single-chain variable fragment (scFv) of the anti-FGF2 3F12E7 monoclonal antibody

**DOI:** 10.1038/s41598-020-80746-8

**Published:** 2021-01-14

**Authors:** Rodrigo Barbosa de Aguiar, Tábata de Almeida da Silva, Bruno Andrade Costa, Marcelo Ferreira Marcondes Machado, Renata Yoshiko Yamada, Camila Braggion, Kátia Regina Perez, Marcelo Alves Silva Mori, Vitor Oliveira, Jane Zveiter de Moraes

**Affiliations:** 1 Department of Biophysics, Escola Paulista de Medicina, Universidade Federal de São Paulo, Rua Três de Maio, 100 – Vila Clementino, São Paulo, SP CEP 04044-020 Brazil; 2 Instituto de Biologia, Universidade Estadual de Campinas, Campinas, Brazil

**Keywords:** Proteins, Immunotherapy

## Abstract

Single-chain variable fragments (scFvs) are small-sized artificial constructs composed of the immunoglobulin heavy and light chain variable regions connected by a peptide linker. We have previously described an anti-fibroblast growth factor 2 (FGF2) immunoglobulin G (IgG) monoclonal antibody (mAb), named 3F12E7, with notable antitumor potential revealed by preclinical assays. FGF2 is a known angiogenesis-associated molecule implicated in tumor progression. In this report, we describe a recombinant scFv format for the 3F12E7 mAb. The results demonstrate that the generated 3F12E7 scFv, although prone to aggregation, comprises an active anti-FGF2 product that contains monomers and small oligomers. Functionally, the 3F12E7 scFv preparations specifically recognize FGF2 and inhibit tumor growth similar to the corresponding full-length IgG counterpart in an experimental model. In silico molecular analysis provided insights into the aggregation propensity and the antigen-recognition by scFv units. Antigen-binding determinants were predicted outside the most aggregation-prone hotspots. Overall, our experimental and prediction dataset describes an scFv scaffold for the 3F12E7 mAb and also provides insights to further engineer non-aggregated anti-FGF2 scFv-based tools for therapeutic and research purposes.

## Introduction

Advances in recombinant antibody technology led to the development of a large variety of engineered monoclonal antibody (mAb) constructions differing in pharmacokinetic and binding properties for research, diagnostic, and therapeutic applications. Among the recombinant antibody formats, there is the single-chain variable fragment (scFv)^1^. ScFvs consist of artificial constructs composed of the variable regions of the heavy (V_H_) and light (V_L_) immunoglobulin chains connected by a flexible peptide linker in a way to retain the antigen-binding properties of the corresponding IgG antibody^2^. The scFvs are often produced with high yield using bacterial expression systems^3^, which comes as an advantage over the production of full-sized IgGs by hybridoma cells. Also, the expected small molecular size for such recombinant constructions may imply higher diffusion inside tumors and blood clearance^1, 3^. Several scFvs have been described to target tumors, with some of them already approved for clinical use^4,5^.

To target tumor growth, our research group previously generated an immunoglobulin G (IgG) monoclonal antibody (mAb) directed against the fibroblast growth factor 2 (FGF2)^6^, based on the conventional hybridoma technology^7^. FGF2 is a key angiogenesis-related factor implicated in the development and progression of several tumors^8–10^. The biological effects of FGF2 are exerted through the formation of ternary FGF2-heparin-FGF receptor complexes in several cells in the tumor microenvironment, including endothelial and neoplastic cells^9,11,12^.

Our anti-FGF2 IgG mAb, named 3F12E7, showed promising results in experimental approaches. It was demonstrated to successfully reduce tumor blood vessel density and to inhibit tumor growth and metastasis, as previously described^6^. However, despite the promising data, this full-length IgG mAb failed to inhibit the growth of already established tumors, which encouraged us to design new small-sized agents to target FGF2.

In this report, we describe an scFv construct generated by the fusion of the V_H_ and V_L_ domains of the 3F12E7 mAb. The functional activity of the 3F12E7 scFv was evaluated considering its aggregation and binding properties. Our results revealed a soluble anti-FGF2 product that, although prone to aggregation, contains monomers and small oligomers. The obtained scFv succeeded in specifically recognizing FGF2 in tumor extracts and inhibiting experimental tumor growth similar to the corresponding full-length IgG antibody counterpart. Also, in-silico predictions provide useful insights to understand the antigen-recognition by scFv units and to further engineer non-aggregated scFv-based tools for therapeutic and experimental applications.

## Results

### Construction and functional characterization of anti-FGF2 3F12E7 scFv

**Figure 1:**
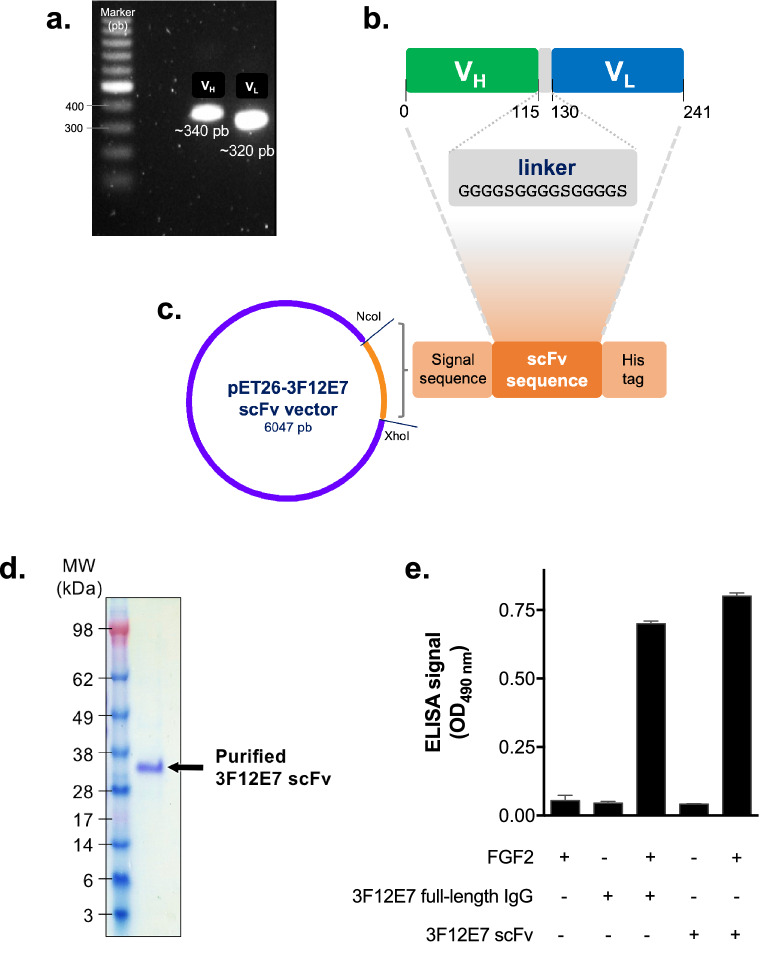
Generation of 3F12E7 anti-FGF2 scFv. (**a**) Agarose gel electrophoresis of PCR amplified products of V_L_ and V_H_ genes from 3F12E7 hybridoma cells. (**b**) Design of the 3F12E7 scFv construct, formed by V_H_ and V_L_ amino acid sequences attached by a flexible peptide linker [(G_4_S)_3_]. (**c**) Schematic representation of the pET26-based vector used for the expression of 3F12E7 scFv in *E. coli*. The insert codes the scFv linked to a signal sequence and a C-terminal 6 × His tag. (**d**) SDS-PAGE analysis of the affinity-purified 3F12E7 scFv, under denaturing and reducing conditions. Gel protein content was detected by Coomassie blue staining. (**e**) Binding of 3F12E7 scFv to FGF2 was accessed by ELISA. 3F12E7 full-length IgG was used as a positive control.

To construct the scFv form of the anti-FGF2 3F12E7 mAb, the DNA segments encoding the antibody heavy (V_H_) and light (V_L_) variable chains were amplified from the cDNA of 3F12E7 hybridoma cells (Fig. 1a) and further sequenced. Blast analysis revealed high homology (> 98%) of the detected V_H_ and V_L_ domains with known mouse immunoglobulin sequences. The scFv was designed in the V_H_-linker-V_L_ orientation^13^, using [(Gly_4_Ser)_3_] peptide as a linker (Fig. 1b).

In a preliminary experimental evaluation, the 3F12E7 scFv gene construct was found effective in generating a product that binds FGF2. The transient transfection of HEK293 cells with the scFv gene inserted into a pcDNA3.1 vector resulted in the successful expression of an FGF2-binding component, as accessed by immunofluorescence assays and cell-bound ELISA (Supplementary Fig. S1). The cellular expression of scFv was detected indirectly with polyclonal anti-FGF2 antibodies, after cell incubation with recombinant FGF2.

To proceed with the study and obtain a purified scFv, we opted to clone the 3F12E7 scFv gene into pET26b vector, which was further transformed to *E. coli* BL21 (DE3) pLysS cells. The bacterial expression vector is schematically indicated in Fig. 1b,c. The scFv was produced in inclusion bodies, which were further solubilized in urea, purified by FPLC over a His-Trap column, and submitted to a dialysis-based refolding procedure, as described in “Methods” section. Protein purity was confirmed by SDS-PAGE. The 3F12E7 scFv exhibited a single band with an apparent molecular mass between 28 and 38 kDa in gel electrophoresis under denaturing and reducing conditions (Fig. 1d), which is within the expected size for the monomeric form of this protein.

**Figure 2:**
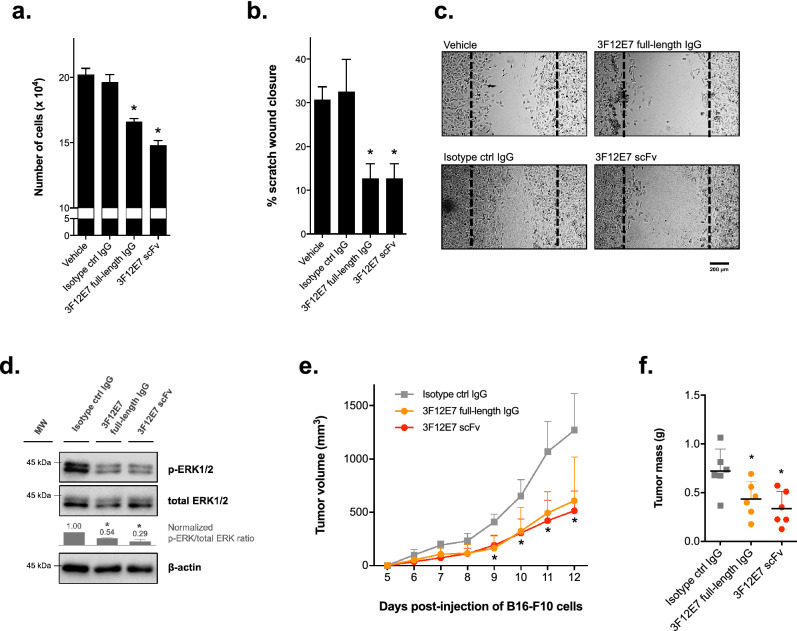
In vitro and in vivo functional effects of 3F12E7 anti-FGF2 scFv. 3F12E7 scFv reduces in vitro endothelial cell proliferation (**a**) and migration (**b**). Cells were incubated with 10 µg/mL of the indicated mAbs. No difference was detected between 3F12E7 scFv and 3F12E7 full-length IgG groups. Cell proliferation and migration were accessed by trypan blue exclusion and scratch assays, respectively. Representative micrographs of the scratch assay are on (**c**). Dashed lines indicate original wound edges. Scale bar, 200 µm. *P < 0.05 compared to isotype and vehicle controls; *one-way* ANOVA/Bonferroni’s post-test. (**d**) Immunoblotting analyses of ERK1/2 phosphorylation in HUVEC after 48-h incubation with the indicated mAbs (50 µg/mL). β-actin was used as loading control. Graph shows the quantitative densitometry of the immunoblot results. Data are mean ± s.d. of the relative intensity of the bands, normalized to that of isotype ctrl IgG group, from two independent assays. The full-length image scans and the result of an additional independent assay are provided in Supplementary Fig. S4a. (**e**, **f**) 3F12E7 scFv reduces xenograft tumor growth similarly to 3F12E7 full-length IgG mAb. (**e**) Tumor growth curve. (**f**) Excised tumor mass on day 12. Treatment started four days after subcutaneous injection of B16-F10 cells. Result (mean ± s.d.) is representative of two independent experiments. Experimental groups: isotype control full-length IgG antibody, n = 6; 3F12E7 full-length IgG mAb, n = 6; 3F12E7 scFv, n = 6. *P < 0.05 compared with isotype control group; *one-way* ANOVA/Bonferroni’s post-test.

The binding of the affinity-purified 3F12E7 scFv to FGF2 was confirmed by ELISA analysis (Fig. 1e). The functional activity of the generated scFv was further assessed by in vitro experiments with endothelial cells. Trypan blue exclusion assays revealed that HUVEC exposure to 10 µg/mL 3F12E7 scFv significantly reduced the number of viable cells compared to that in vehicle and irrelevant IgG groups (Fig. 2a). Such result is similar to the achieved with the 3F12E7 full-length IgG mAb. In the same trend, HUVEC incubated with 3F12E7 scFv (10 µg/mL) for 48 h shows attenuated cell migration in monolayer scratch assay (Fig. 2b,c) and reduced phosphorylation of ERK1/2 (Fig. 2d; Supplementary Fig. S4a).

The antitumor effect of 3F12E7 anti-FGF2 scFv was evaluated in the B16-F10 experimental tumor model. Tumor-bearing mice received 3F12E7 mAb in its full-length or scFv format (or IgG control) every two days. Tumor growth curves and the tumor mass on day 12 are provided in Fig. 2e,f. The 3F12E7 scFv was as effective in reducing tumor growth as the corresponding full-length IgG. All these findings were obtained using 0.22 µm-filtered scFv samples.

### Aggregation state and FGF2 specificity of the 3F12E7 scFv

**Figure 3:**
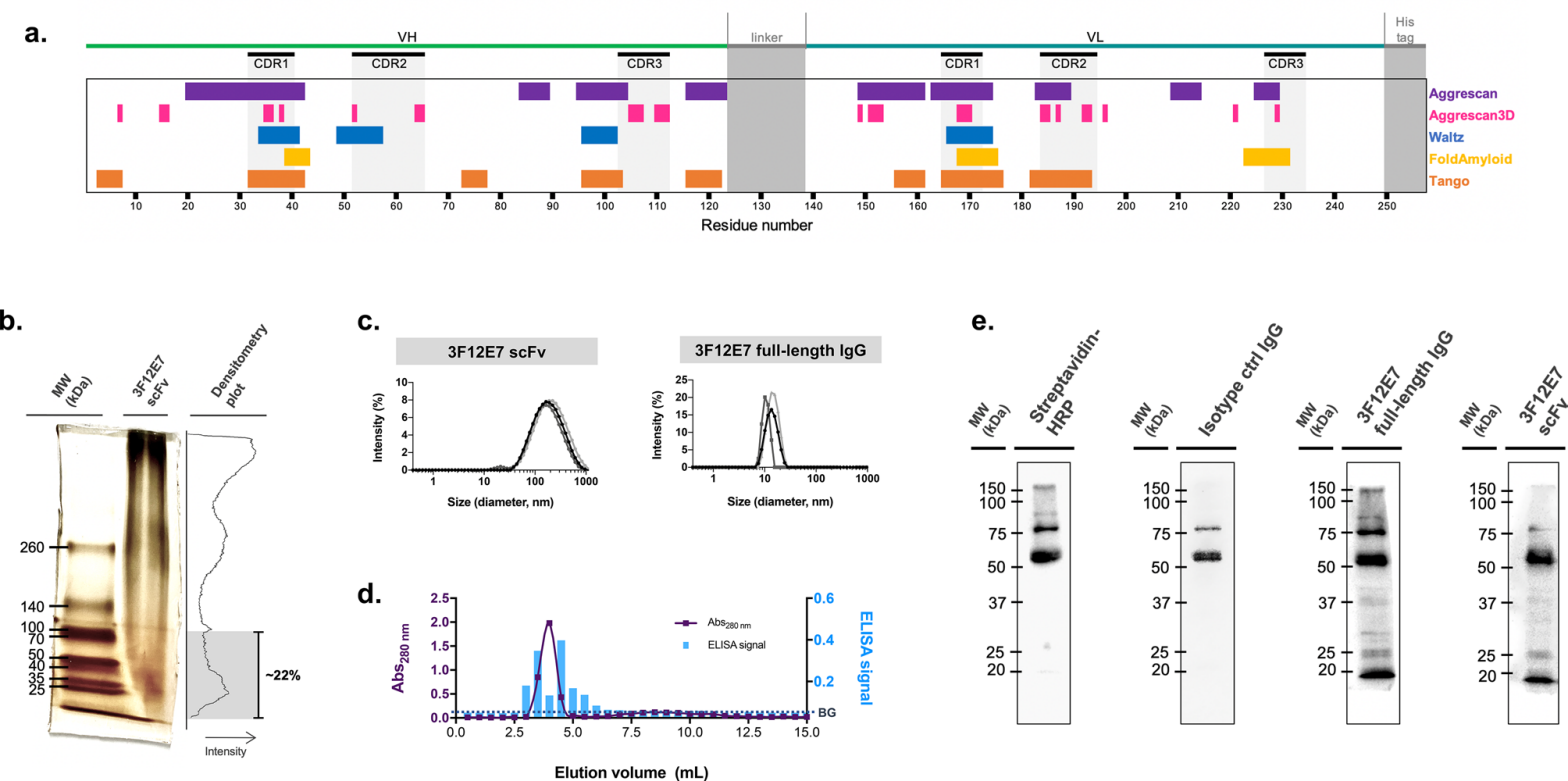
Analysis of the 3F12E7 scFv aggregation state and activity. (**a**) Evaluation of aggregation propensity of 3F12E7 scFv using Aggrescan3D web server and sequence-based predictors. Amino acid residues (numbered according to primary structure) that had positive scores are indicated in different colors for each predictor: Aggrescan (purple); Aggrescan3D (magenta); Waltz (blue); FoldAmyloid (yellow); Tango (orange). (**b**) Oligomeric profile of the 3F12E7 scFv, as assessed by blue native gel polyacrylamide electrophoresis (BN-PAGE). Gel was stained with silver nitrate. Graph on the right shows the corresponding densitometry analysis. (**c**) Dynamic light scattering (DLS) analysis for 3F12E7 scFv (left) and full-length IgG mAb (right). Average particle size results for each mAb are expressed by signal intensity. Each line denotes data obtained for the 3 independent samples. (**d**) Analysis of 3F12E7 scFv by size-exclusion chromatography (on a PD-10 column) and the binding of the obtained fractions to FGF2, as assessed by ELISA. Dashed line indicates ELISA background signal (BG). (**e**) Immunoblotting analysis of the 3F12E7 scFv reactivity to B16-F10 tumor protein extracts. For that, antibodies were labeled with biotin and detected with horseradish peroxidase (HRP)-streptavidin. These immunoblot images are also provided in Supplementary Fig. S4b. MW, molecular weight (kDa).

Production of functional scFv in bacteria is known to be challenging due to the frequent protein accumulation within inclusion bodies and the formation of kinetically trapped misfolded units^14^, which accentuates the property of particular scFv constructs to aggregate. Considering the protein primary structure, different web-based servers (Aggrescan, Aggrescan3D, Waltz, FoldAmyloid, and Tango) were used to predict the aggregation-prone segments of the 3F12E7 scFv. Figure 3a illustrates the localization of these segments (mainly referring to hydrophobic stretches) in the scFv amino acid sequence. Most of the aggregation hotspots were predicted near the CDRs of V_H_ and V_L_ domains. CDRs were determined using the Paratome web server.

The aggregation state of the 3F12E7 scFv was assessed by native gel electrophoresis (BN-PAGE) and dynamic light scattering (DLS) analysis. BN-PAGE data indicate monomer and small oligomers in the scFv preparation. The representative BN-PAGE profile in Fig. 3b shows that ~ 22% of the scFv content has less than 100 kDa. On the other hand, the detection of soluble high molecular complexes should not be ignored, which is in line with the DLS findings indicating the presence of large aggregated material (hydrodynamic size above 100 nm) in the 0.22 µm-filtered scFv preparation (Fig. 3c). For comparison, the average particle diameter of 3F12E7 full-length IgG was 11 nm.

Size-exclusion chromatographic (SEC) analysis of soluble 3F12E7 scFv samples (on a PD-10 column) revealed that the initial eluted fractions with 280-nm absorbance have different FGF2 binding properties, as indicated in Fig. 3d. Despite the aggregation status of the obtained scFv product, it is possible that the detected antitumor effect following 3F12E7 scFv treatment is not just due to potential nonspecific effects in response to aggregated protein structures. The administration of soluble heat-aggregated IgG complexes did not lead to reduced tumor growth, compared to the obtained with the isotype IgG control (Supplementary Fig. S2), in the employed in vivo therapeutic intervention. To note, the isotype IgG control preparations used in all assays also contain large structures, compared to the folded monomer’s expected size, although this occurs in a lower proportion than in 3F12E7 scFv (Supplementary Fig. S2a, c).

The 3F12E7 scFv was found specific to FGF2. Immunoblotting assays performed with B16-F10 tumor extracts showed that biotinylated 3F12E7 scFv exhibited the same protein labeling pattern as that of the full-length IgG counterpart. An ~ 18-kDa band is detected in blots incubated with both formats of the 3F12E7 mAb (Fig. 3e; Supplementary Fig. S4b). The biotinylated scFv preparation comprises an oligomer-monomer mixture, as indicated in Supplementary Fig. S3. The most evident bands exhibited an apparent molecular mass compatible with monomer, dimers, and octamers of the scFv construct. Also, the SEC elution fractions of the biotinylated preparation show an FGF2-binding profile similar to that detected with unlabeled scFv.

### Prediction of 3F12E7 scFv interaction with FGF2

**Figure 4:**
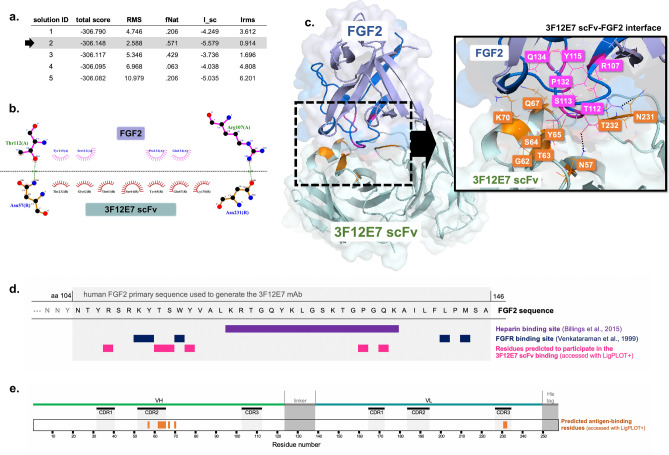
Prediction of 3F12E7 scFv binding to FGF2. (**a**) Metrics of the top-ranked 5 poses found by molecular docking between FGF2 (PDB ID: 1bfg) and 3F12E7 scFv with the Rosetta server. Based on CAPRI criteria, solution ID #2 was chosen for interaction analyses. (**b**) LIGPLOT^+^ diagram of the residues interacting across the 3F12E7 scFv-FGF2 interface. 3F12E7 scFv and FGF2 residues are labeled brown and magenta, respectively. Hydrophobic interactions are represented by arc with spokes and hydrogen bonds are indicated by dashed green lines. Hydrogen bonds were detected between R107 and T112 of FGF2 and N57 and N231 of the scFv entity, respectively. (**c**) 3D representation of the 3F12E7 scFv-FGF2 putative complex. FGF2 and 3F12E7 scFv residues found by LIGPLOT^+^ analyses are labeled magenta and orange, respectively. Hydrogen bonds are indicated by dashed lines. In dark blue, it is highlighted the FGF2 amino acid sequence (residues 104–146) used for the generation of the anti-FGF2 3F12E7 mAb, as reported^6^. Within this segment, the residues predicted to interact with 3F12E7 scFv are schematically indicated on (**d**), along with the heparin and FGFR binding sites. (**e**) Localization of the putative FGF2-contacting residues and the Paratome CDRs in the 3F12E7 scFv sequence.

In silico molecular analyses provided insights into the interaction between the 3F12E7 scFv and its ligand, FGF2 (PDB ID: 1bfg), using for that a homology model of the scFv assembly in a monomeric closed state. Docking studies were performed with the Rosetta server and metrics of the top 5-scored decoys are presented in Fig. 4a. The output that best fits the CAPRI quality scheme was chosen for further identification of the anchoring spots on the protein surface. The selected decoy showed interface energy score (I_sc) of − 5.579, fraction of native contacts (Fnat) of 0.571, and total score of − 306.148.

The analysis of the 3F12E7 scFv monomer-FGF2 putative interface and the 3D cartoon representation of the docked complex are depicted in Fig. 4b,c. The 3F12E7 scFv module was predicted to contact the FGF2 residues R107, T112, S113, T115, P132, and N134 (Fig. 4b), which are located within or near the heparin- or FGFR-binding domains in FGF2 (Fig. 4d). The 3F12E7 scFv residues implicated in such binding are positioned mainly at V_H_-CDR2 and V_L_-CDR3, as indicated in Fig. 4e.

## Discussion

Herein we report the construction and the initial characterization of an scFv format of the already described anti-FGF2 3F12E7 mAb^6^. Our molecular construct follows a traditional scFv scheme comprising the V_H_ and V_L_ segments of the IgG counterpart. These domains were joined by a 15-residue glycine-serine linker, used in many of the reported scFvs^13, 15–18^, leading to an artificial V_H_-V_L_ arrangement – named 3F12E7 scFv – whose monomer molecular mass is around 35 kDa.

The 3F12E7 scFv was characterized from both functional and colloidal perspectives. The provided experimental results demonstrate the scFv effectiveness in binding FGF2 and inhibiting the growth of B16-F10 experimental tumors, known to be dependent on the FGF2-mediated signaling^6,19,20^. These findings are similar to those achieved with the corresponding full-length IgG mAb and come from a product containing scFv monomer and small oligomers, as assessed by native electrophoresis (BN-PAGE). But regarding the 3F12E7 scFv colloidal profile, what attracts attention is the high soluble aggregate content. Based on dynamic light scattering (DLS) measurements, some of the soluble scFv particles surpasses 100 nm in hydrodynamic diameter, which is several times greater than that detected for a full-length IgG antibody.

The 3F12E7 scFv aggregation potentially reflects aspects related to the employed protein expression and refolding strategy. Large-scale production of scFvs in bacterial expression systems, although practical and time efficient, often leads to a product containing aggregates^14^. This may be significantly boosted by the particular propensity of the designed scFv to aggregate. Our in-silico analyses pointed several aggregation hotspots within the 3F12E7 scFv amino acid sequence. This feature should not be considered exclusive to our construction, since high-molecular-mass multimers have already been found in previously described scFv preparations^21–23^.

An scFv product composed of large soluble aggregate particles naturally raises concerns about its biological applicability. As an additional complicating factor, the high protein concentration formulations typically used for in vivo applications may favor the formation of aggregates^24^. Notwithstanding that, the 3F12E7 scFv revealed specific to FGF2, as assessed by immunoblotting assays. Also, SEC analysis of such soluble scFv product showed that even the initial protein elution fractions, supposedly enriched by large aggregate entities, bind to FGF2 without any important cross-reactivity to albumin. It should be further noted that the antigen-binding profile of these SEC fractions provides evidence that 3F12E7 scFv multimeric species have distinct anti-FGF2 properties. Two nonconsecutive fractions are underscored by their relatively high reactivity to FGF2, compared to the other evaluated samples. It is possible that such characteristic comes from a particular oligomeric content exhibiting multivalence, with higher avidity over the monomeric counterpart, which is consistent with the already described for other scFvs^25,26^.

Although misfolded forms are typically not active and prone to irreversible aggregation, we cannot discard that denatured antibody complexes may retain a fraction of functionally active fragments. Following SEC separation of biotinylated 3F12E7 scFv, an analysis of the initial protein eluates through BN-PAGE points out strong monomer-related bands in all these fractions. It is possible that monomers weakly anchored to denatured core units were released during electrophoretic separation, contributing in part to the obtained result. Also, we cannot rule out that the biotin labeling reaction, performed after the protein purification and refolding steps, could have impaired, in some way, the stability of the scFv complexes. What our findings collectively demonstrate, though, is that, despite being densely populated by soluble aggregated components, both the unlabeled and the biotinylated scFv materials are anti-FGF2 active and comprise a structurally and functionally heterogeneous molecular repertoire that includes monomeric and small oligomeric conformers. It should be noted that, although DLS data do not reveal particles compatible with small scFv units, this may be due to the light scattering by large structures. According to Rayleigh’s approximation, the intensity of scattering is proportional to the sixth power of a particle diameter^27^.

Our exploratory in-silico analyses additionally provide mechanistic insights into the antigen–antibody interaction. The 3F12E7 scFv was predicted to contact non-linear stretches within the FGF2 surface, which englobes the heparin and the receptor-binding sites. The inhibition of such FGF2 function-related domains by the scFv construct possibly contributed to the detected in vitro and in vivo effects. It is known that the interaction of FGF2 to both its cognate receptor and heparin accounts for the FGF2-mediated signal transduction^11,12^. It is worth noting that our computational predictions are in line with the previous finding that the full-length IgG format of the 3F12E7 mAb inhibits the binding of FGF2 to FGFR^6^. Also, it is interesting to note that, consistent with previous reports^28,29^, most of the 3F12E7 scFv aggregation-prone motifs were predicted within CDR loops. Despite that, the CDR1 domain, predicted to concentrate most of the aggregation-prone residues in both heavy- and light-chain antibody segments, potentially does not have a critical role in FGF2 binding. In-silico analysis of the putative scFv-FGF2 interface revealed that most of the antibody determinants involved in antigen recognition are located within the heavy-chain CDR2 and the light-chain CDR3, whose residues are less prone to aggregation.

Overall, our results describe and discuss a promising scFv scaffold of the 3F12E7 mAb. While the detected colloidal features are away from the desired for in vivo interventions^3^, the demonstrated specificity to FGF2 put the employed scFv design as a potential template to be further used to engineer a monomeric product for therapeutic purposes and also for addressing the significance of FGF2 inhibition on tumor growth.

## Methods

### Mammalian cell culture

Human Umbilical Vein Endothelial Cell (HUVEC), HEK293T, and B16-F10 cells, all of them obtained from ATCC (USA), were cultured in RPMI-1640 medium (Sigma-Aldrich, USA) with 10% fetal bovine serum (FBS; Gibco BRL, USA). 3F12E7^6^ and 1F5H2^30^ hybridoma cells were cultured in the same medium with the addition of 50 μM 2-mercaptoethanol. All cells were maintained at 37 °C in 5% CO_2_ and were checked for *Mycoplasma* contamination.

### Design of the 3F12E7 scFv

Total RNA of 3F12E7 hybridoma cells was isolated with TRIzol (Thermo, USA) and the cDNA was synthesized using a cDNA reverse transcription kit (Applied Biosystems, USA). The V_H_ and V_L_ DNA fragments were amplified by PCR using 1 µM primers, 40 U/mL Vent polymerase (NEB BioLab), and 0.2 mM dNTP, as described previously^13,31,32^. For V_H_ region, the forward primer is VH1FOR-2 (primer group A) and the back primer, VH1BACK (primer group B). For V_L_ chain, a mix of four different primers (MJK1FONX, MJK2FONX, MJK4FONX, and MJK5FONX) (primer group C) to the J gene segments encoding most of the mouse V kappa families was used as forward primer. VK2BACK (primer group D), common to the four kappa light chains, was used as back primer^32^. Primer sequences are listed in Table 1.

**Table 1:** Sequence of synthetic oligonucleotide primers used for PCR amplification.

Group	Primer type	Sequence
A	Forward	**VH1FOR-2:** 5′-TGA GGA GAC GGT GAC CGT GGT CCC TTG GCC CC-3′
B	Reverse	**VH1BACK:** 5′-AG GT{G/C} {A/C}A{A/G} CTG CAG {G/C}AG TC{A/T} GG-3′
C	Forward	**MJK1FONX:** 5′ -CCG TTT GAT TTC CAG CTT GGT GCC-3′; **MJK2FONX:** 5′ -CCG TTT TAT TTC CAG CTT GGT CCC-3′; **MJK4FONX:** 5′ -CCG TTT TAT TTC CAA CTT TGT CCC-3′; **MJK5FONX:** 5′-CCG TTT CAG CTC CAG CTT GGT CCC-3′
D	Reverse	**VK2BACK:** 5′-GAC ATT GAG CTC ACC CAG TCT CCA-3′
E	Forward	5′-GGT GGT CCA TGG ATC TAA TGG CCC AGG TGA AAC TGC AGG-3'
F	Reverse	5′-CAA CAA CTC GAG TGC GGC CGC CCG TTT GAT TTC CAG CTT-3'

PCR was run at 94 °C/3 min, followed by 30 cycles of 94 °C/1 min (denaturation), 56 °C/1 min (annealing), and 72 °C/1 min (elongation), with a final 5 min reaction at 72 °C. The amplified products from the V_H_ (with 340 bp) and V_L_ (with 325 bp) region were separated on 1% agarose gel, purified with GeneClean kit (BIO 101 Inc., USA), and further analyzed by an automated DNA sequencer (ABI377; Applied Biosystems, USA). Paratome web server (available at http://www.ofranlab.org/paratome/) was used to predict the complementary-determining regions (CDRs) of 3F12E7 scFv, based on the primary sequence of the heavy and light variable chains^33^.

The 3F12E7 scFv was constructed in a V_H_-linker-V_L_ format, as described previously^13^, using peptide [(Gly_4_Ser)_3_] peptide as a linker. The 3F12E7 scFv gene was acquired from GenScript gene synthesis service (USA) into the pcDNA3.1 expression vector. This construct, named pcDNA3.1-3F12E7 scFv, contains the scFv-coding sequence inserted between *Hind*III and *Xba*I restriction sites. The plasmid vector was digested with *Hind*III and *Xba*I and the DNA encoding scFv was confirmed by DNA sequencing. The PCR amplification was carried out in the same conditions described above.

### Expression of 3F12E7 scFv in bacteria

To obtain the 3F12E7 scFv protein, the related gene, inserted into pcDNA3.1-3F12E7 scFv plasmid, was cloned into the pET26b vector between *Nco*l and *Xho*I restriction sites, using primers E and F (Table 1). This construction, named pET26b-3F12E7 scFv, was sequence-verified and planned to code the 3F12E7 scFv with an N-terminal signal sequence (MKYLLPTAAAGLLLLAAQPAMA) and a C-terminal poly-histidine tag (LEHHHHHH).

The pET26b-3F12E7 scFv vector was transformed into *E. coli* BL21 (DE3) pLysS (Novagen), used as an expression host. A single colony of transformed *E. coli* cells was incubated overnight at 37 ºC in 10 mL of Luria–Bertani (LB) medium containing kanamycin (50 µg/mL) and chloramphenicol (50 µg/mL), with shaking at 150 rpm. Pre-culture was diluted 200 × in 1.5 L of fresh LB medium and incubated until the culture density reaches an OD_600_ between 0.6 and 0.8. The expression of 3F12E7 scFv was induced by 0.5 mM isopropyl β-D-1-thiogalactopyranoside (IPTG) for 4 h. Then, bacterial cells were harvested by centrifugation at 6000 rpm for 10 min and the cell pellet was further used for scFv purification.

### 3F12E7 scFv purification

Bacterial cells expressing 3F12E7 scFv were suspended in TB buffer (50 mM Tris, pH 7.5) and disrupted by using French Pressure Cell Press (1.6 MPa). The resulting lysate was centrifuged at 10,000 rpm for 10 min. The insoluble fraction was washed twice with TB buffer, incubated with solubilization buffer (50 mM Tris, 300 mM NaCl, 8 M urea, pH 7.5) for 2 h, and then centrifuged at 10,000 rpm. The supernatant was loaded, at a flow rate of 2.5 mL/min, on a Ni-Sepharose high-performance column (GE Healthcare) equilibrated with solubilization buffer and attached to an FPLC system (ÄKTA; GE, USA). Purified scFv was recovered with 200 mM imidazole in elution buffer (50 mM Tris, 300 mM NaCl, 8 M urea, 500 mM imidazole, pH 7.5) and was further analyzed by SDS-PAGE. Protein refolding procedure was performed by 48-h dialysis against water at 4 °C, with water change every 12 h. Samples were filtered through a 0.22-µm filter prior to their in vitro and in vivo application.

### Full-length IgG antibody purification

Anti-FGF2 3F12E7^6^ and CEA-binding 1F5H2^30^ full-length IgG1 mAbs were purified on a protein G Sepharose (GE Healthcare, USA) column. 1F5H2 mAb was used as irrelevant antibody in all performed experiments. Antibody concentration was determined by measuring the sample absorbance at 280 nm (Nanodrop; Thermo Scientific, USA).

### Enzyme immunoassay (ELISA) studies

3F12E7 scFv binding to FGF2 was initially verified by sandwich ELISA. Briefly, 96-well plates were coated overnight at 4 °C with 5 µg/mL of (i) 3F12E7 scFv; (ii) 3F12E7 full-length IgG mAb (positive control); or (iii) irrelevant full-length IgG (negative control). The wells were blocked with 1% bovine serum albumin (BSA; Sigma, USA) in phosphate-buffered saline (PBS) and, then, incubated (or not) with 50 ng/mL recombinant FGF2 (PeproTech Inc., USA). Subsequently, wells were incubated with polyclonal anti-FGF2 antibody (1:10,000; Sigma, USA) and then with biotin-conjugated secondary antibody (1:5000; Sigma, USA), both diluted in 0.1% BSA containing 0.1% Tween-20 (Sigma, USA) (PBST).

The binding of size exclusion chromatographic elution fractions of 3F12E7 scFv to FGF2 was detected by direct ELISA. For that, wells were coated overnight at 4 °C with 3F12E7 scFv (5 µg/mL). After blocking with 1% BSA in PBS, wells were incubated with FGF2 (PeproTech Inc., USA) labeled to biotin. FGF2 biotinylation was performed using the EZ-Link Sulfo-NHS-Biotin kit (Thermo Scientific, USA).

In all cases, the wells were washed three times with PBST between each step. The reactions were revealed with horseradish peroxidase (HRP)-streptavidin (1:2000; Sigma, USA) and *o*-phenylenediamine (OPD; Sigma, USA) substrate. Absorbance values were read at 490 nm.

### Immunoblotting

3F12E7 scFv immunoreactivity to murine tumors was evaluated on B16-F10 melanoma protein extracts separated on 15% SDS–polyacrylamide gel and further transferred to PVDF membrane (Millipore, USA). After blocking, blots were probed overnight at 4 °C with 2 µg/mL of the following biotin-labeled mAbs: (i) 3F12E7 scFv; (ii) 3F12E7 full-length IgG; or (iii) irrelevant full-length IgG. Antibody biotinylation was carried out using the EZ-Link Sulfo-NHS-Biotin kit (Thermo Scientific, USA). Membrane-bound antibodies were detected with HRP-streptavidin (1:1000; Sigma, USA).

ERK1/2 phosphorylation was assessed in extracts of HUVEC incubated for 48 h with 10 or 50 µg/mL 3F12E7 scFv. 3F12E7 full-length IgG and irrelevant full-length IgG mAbs (10 or 50 µg/mL) were used as controls. For that, protein cell lysates were resolved on 10% SDS–polyacrylamide gels, transferred to PVDF membranes and the blots were probed overnight at 4 °C with 1:2000-diluted anti-phospho-p44/42 MAPK (phospho-ERK1/2 at Thr202/Tyr204; #4376, Cell Signaling, USA) and anti-p44/42 MAPK (total ERK1/2; #9102, Cell Signaling, USA) antibodies. β-actin (1:1000; ab8227, Abcam, USA) was used as loading control. Then, the membranes were washed with TBST and incubated with HRP-conjugated secondary antibody (1:7000; ab6721, Abcam, USA) for 1 h at room temperature.

In all cases, 5% BSA in PBST was used as blocking buffer. Protein extracts were obtained with lysis buffer (50 mM Tris, pH 8.0, 150 mM NaCl, 0.5% Triton X-100, 10% glycerol, and protease inhibitors) and the protein content was determined by Bradford assay. Blots were revealed with ECL reagent (Thermo Scientific, USA) and chemiluminescence was captured using an Alliance imaging system (UVITEC Cambridge, UK). Bands were quantified using ImageJ software (NIH, USA).

### Dynamic light scattering (DLS)

DLS studies were performed with Zetasizer Nano ZS equipment (Malvern, UK) using quartz cuvettes. For that, the antibodies were diluted in PBS to a final concentration of 50 µg/mL. The light scattering measurements were detected at 660 nm and data were collected at 25 °C using a solvent refractive index of 1.333^34^. DLS of each antibody was measured in triplicates.

### Trypan blue exclusion assay

HUVECs (1.5 × 10^3^ cells/100 μL/well) were plated on 96-well plates and, 24 h later, incubated with 10 µg/mL 3F12E7 scFv. 3F12E7 full-length IgG and irrelevant full-length IgG mAbs (10 µg/mL) were used as controls. After 72 h, cells were harvested and counted using a hemocytometer. The number of viable cells was determined by the trypan blue exclusion. The experiment was performed in quadruplicate and repeated three times.

### In vitro scratch assay

HUVECs (0.75 × 10^5^ cells/500 μL/well) were cultured on 24-well plates until confluence. Then, a scratch was made through the cell monolayer using a 200 µL pipette tip. Cells were washed twice with PBS and incubated with 10 µg/mL 3F12E7 scFv. 3F12E7 full-length IgG and irrelevant full-length IgG mAbs (10 µg/mL) were used as controls. The scratch area was photographed over 48 h using AxioObserver Z1 imaging system (Zeiss, Germany), and the percentage of the initial scratch area was measured using ImageJ software (NIH, USA). Experiment performed in triplicates.

### Animals

C57BL/6 male mice (7–8-week-old) were obtained from the animal facility of “Centro de Desenvolvimento de Modelos Experimentais para Medicina e Biologia” (CEDEME/UNIFESP, Brazil). Animals were maintained with water ad libitum in a temperature- and humidity-controlled room (12:12-h light:dark cycle). At the end of the experiments, mice were euthanized with anesthetic overdose (ketamine and xylazine), according to the AVMA Guidelines for the Euthanasia of Animals^35^. All procedures, approved by the research ethics committee of the Universidade Federal de São Paulo (protocol number 2303230217), were performed according to the guidelines of the National Institutes of Health (NIH).

### In vivo* tumor growth*

The antitumor effect of anti-FGF2 3F12E7 scFv was evaluated using an experimental melanoma model. Mice were subcutaneously implanted with 5 × 10^5^ B16-F10 cells in the left flank and, four days later, started receiving intraperitoneally 2 mg/kg 3F12E7 scFv. As control groups, mice received 2 mg/kg of 3F12E7 full-length IgG mAb or irrelevant full-length IgG. Treatment was performed once every two days, for 10 days. N = 7 mice/group. Tumor volume was daily monitored and calculated as follows: tumor volume [mm^3^] = 0.52 × (tumor length) [mm] × (tumor perpendicular width)^2^ [mm^2^].

### Prediction of aggregation hotspots

The aggregation propensity of 3F12E7 scFv was predicted using the following tools: Aggrescan^36^, Aggrescan3D^37^, Waltz^38^, FoldAmyloid^39^, and Tango^40^. The FASTA format sequence of 3F12E7 scFv was used as the input for Aggrescan, Waltz, FoldAmyloid, and Tango. For Aggrescan3D, it was used the scFv 3D structure generated by SWISS-MODEL (http://swissmodel.expasy.org). The input parameters for each predictor are listed in Table 2.

**Table 2:** Input parameters for each method employed to predict amyloidogenic regions within 3F12E7 scFv amino acid sequence.

Method	Input parameters
Aggrescan	Not required
Aggrescan3D	Distance of aggregation: 8 Å
Waltz	Threshold: best overall performance pH: 7
FoldAmyloid	Scale: expected number of contacts 8 Å Averaging frame: 5 Threshold: 21.4
Tango	N-terminus protected: No C-terminus protection: No pH: 7 Temperature: 298.15 K Ionic strength: 0.02

### Blue native polyacrylamide gel electrophoresis

Blue native polyacrylamide gel electrophoresis (BN − PAGE) was performed as described^41^. Antibody samples were mixed with an equal volume of sample buffer containing 62.5 mM Tris–HCl, pH 6.8, 40% glycerol, and 1% Coomassie Brilliant Blue G-250 and analyzed using a 4–15% polyacrylamide gel. The cathode buffer comprised 15 mM Bis–Tris, pH 7.0, 50 mM tricine, and 0.02% Coomassie Brilliant Blue G-250, and the anode buffer contained 50 mM Bis–Tris, pH 7.0. Densitometry analysis of protein bands was performed using ImageJ software (NIH, USA).

### Molecular docking studies

Inter-molecular interaction between 3F12E7 scFv monomer and FGF2 (PDB ID: 1bfg) was analyzed by molecular docking studies. For that, a 3D structural model of 3F12E7 scFv in a monomeric close state was generated using the SWISS-MODEL homology server^42^. Antibody-ligand docking was performed by the Rosetta server and the quality of output models was assessed using CAPRI criteria, based on the interface root mean square deviation (Irms), the interaction score (I_sc), and the fraction of native contacts (Fnat). The best pose with Irms ≤ 1 Å, I_sc ≤ − 5, and Fnat ≥ 0.5 was selected and visualized using PYMOL (v1.7). The 3F12E7 scFv residues interacting with the FGF2 segment (104–146 amino acid residues) used for the generation of the original 3F12E7 full-length IgG mAb were determined with LigPlot^+^ (ver. 1.4) package, using DIMPLOT module and default parameters^43^.

### Statistical analysis

Data are expressed as mean ± s.d. of at least three independent experiments. Statistical differences between groups were evaluated by Student’s *t*-test or by *one-way* ANOVA followed by Bonferroni’s post-test. *P* < 0.05 was considered significant.

## Supplementary Information

Supplementary Information.

## Data Availability

The data that support the findings of this study are available from the corresponding authors upon reasonable request.

## Abbreviations

CDR: Complementarity-determining region
ctrl: Control
FGF2: Fibroblast growth factor 2
HUVEC: Human umbilical vein endothelial cell
IgG: Immunoglobulin G
mAb: Monoclonal antibody
scFv: Single-chain variable fragment
VH: Variable heavy domain
VL: Variable light domain
