# Supplementary material for: Generation and functional characterization of a single-chain variable fragment (scFv) of the anti-FGF2 3F12E7 monoclonal antibody
Source: Sci Rep. 2021 Jan 14;11:1432. doi: 10.1038/s41598-020-80746-8 (PMC7809466; doi:10.1038/s41598-020-80746-8)
Supplement: Supplementary file 1 — Supplementary Information. [file 41598_2020_80746_MOESM1_ESM.pdf]

SUPPLEMENTARY INFORMATION

Generation and functional characterization of a single-chain variable fragment (scFv) of the anti-FGF2 3F12E7 monoclonal antibody

Rodrigo Barbosa de Aguiar[1§*], Tábata de Almeida da Silva[1§], Bruno Andrade Costa[1§], Marcelo Ferreira Marcondes Machado[1], Renata Yoshiko Yamada[1], Camila Braggion[1], Kátia Regina Perez[1], Marcelo Alves da Silva Mori[2], Vitor Oliveira[1], Jane Zveiter de Moraes[1*].

This file contains:

- Supplementary Methods .... Page 2
- Supplementary Figure S1 .... Page 5
- Supplementary Figure S2 .... Page 6
- Supplementary Figure S3 .... Page 7
- Supplementary Figure S4 .... Page 8

SUPPLEMENTARY METHODS

Expression of 3F12E7 scFv in mammalian cells

5 × 10^5 HEK293T cells were grown for 24 h on sterile glass cover slips settled on a 6-well plate. Cells were transfected with 3.5 µg pcDNA3.1-3F12E7 scFv plasmid vector (or empty vector, as a control) using the calcium phosphate co-precipitation method (Kwon et al., 2013) and incubated for 5 h. The scFv expression was analyzed by immunofluorescence and cell-bound ELISA.

Immunofluorescence assay

For immunofluorescence detection of 3F12E7 scFv in HEK293T cells transfected with pcDNA3.1-3F12E7 vector, the cells were fixed in 1% paraformaldehyde and blocked with 1% BSA/PBS. Subsequently, cells were incubated with 100 ng/mL recombinant FGF2 (PeproTech Inc., USA) and with polyclonal anti-FGF2 antibody (1:1,000 diluted; Sigma, USA). The antibody was detected using 1:200-diluted anti-rabbit secondary antibody conjugated to Alexa Fluor 488 (Thermo, USA). Nuclei were stained with DAPI. Images were captured on a Leica TCS SP8 microscope (Leica, Germany) with a 40x objective.

Cell-bound ELISA

HEK293T cells were seeded in 96-well plates and transfected with pcDNA3.1-3F12E7 scFv vector, as described previously. Then, cells were fixed with 1% paraformaldehyde, blocked with 1% BSA/PBS, and incubated (or not) with 100 ng/mL recombinant FGF2 (PeproTech Inc., USA). After washing with PBST, cells were incubated with biotin-conjugated polyclonal anti-FGF2

antibody (1:1,000; Sigma, USA) diluted in 0.1% BSA/PBST. The reaction was revealed with HRP-streptavidin (1:1,000; Sigma, USA) and *o*-phenylenediamine (OPD; Sigma, USA) substrate. Absorbance values were read at 490 nm.

ELISA detection of the binding of biotinylated 3F12E7 scFv to FGF2

96-well plates were coated with FGF2, as described (Parise et al., 2008; de Aguiar et al., 2016). After blocking with 1% BSA in PBS, wells were incubated overnight at 4 ^{o}C with chromatographic elution fractions (on a PD-10 column) of biotin-labeled 3F12E7 scFv (1 µg/mL). 3F12E7 scFv biotinylation was carried out using the EZ-Link Sulfo-NHS-Biotin kit (Thermo Scientific, USA). The antigen-antibody binding was detected using HRP-streptavidin (1:1,000; Sigma, USA) and 3,3',5,5'-tetramethylbenzidine (TMB; Sigma, USA) substrate. The wells were washed three times with PBST between each step. Absorbance values were read at 450 nm after the reaction was stopped.

Preparation of heat-aggregated IgG complexes

Heat-aggregated IgG complexes were produced by incubating the 1F5H2 full-length IgG (de Moraes et al., 1994), used as isotype ctrl IgG in the performed experiments, for 20 min at 63 ^{o}C, as described (Ostreiko et al., 1987). Samples were filtered through a 0.22-µm filter prior to their *in vitro* and *in vivo* application. The soluble IgG aggregates were analyzed by BN-PAGE and DLS assays.

References

de Aguiar RB, Parise CB, Souza CR, et al. Blocking FGF2 with a new specific monoclonal antibody impairs angiogenesis and experimental metastatic melanoma, suggesting a potential role in adjuvant settings. Cancer Lett. 2016;371(2):151-160. doi:10.1016/j.canlet.2015.11.030.

de Moraes JZ, Gesztesi JL, Westermann P, Le Doussal JM, Lopes JD, Mach JP. Anti-idiotypic monoclonal antibody AB3, reacting with the primary antigen (CEA), can localize in human colon-carcinoma xenografts as efficiently as AB1. Int J Cancer. 1994 May 15;57(4):586-91.

Kwon M, Firestein BL. DNA transfection: calcium phosphate method. Methods Mol Biol. 2013;1018:107-10. doi: 10.1007/978-1-62703-444-9_10.

Ostreiko KK, Tumanova IA, Sykulev YuK. Production and characterization of heat-aggregated IgG complexes with pre-determined molecular masses: light-scattering study. Immunol Lett. 1987;15(4):311-316. doi:10.1016/0165-2478(87)90134-9.

Parise CB, Lisboa B, Takeshita D, Sacramento CB, de Moraes JZ, Han SW. Humoral immune response after genetic immunization is consistently improved by electroporation. Vaccine. 2008;26(31):3812-3817. doi:10.1016/j.vaccine.2008.05.029.

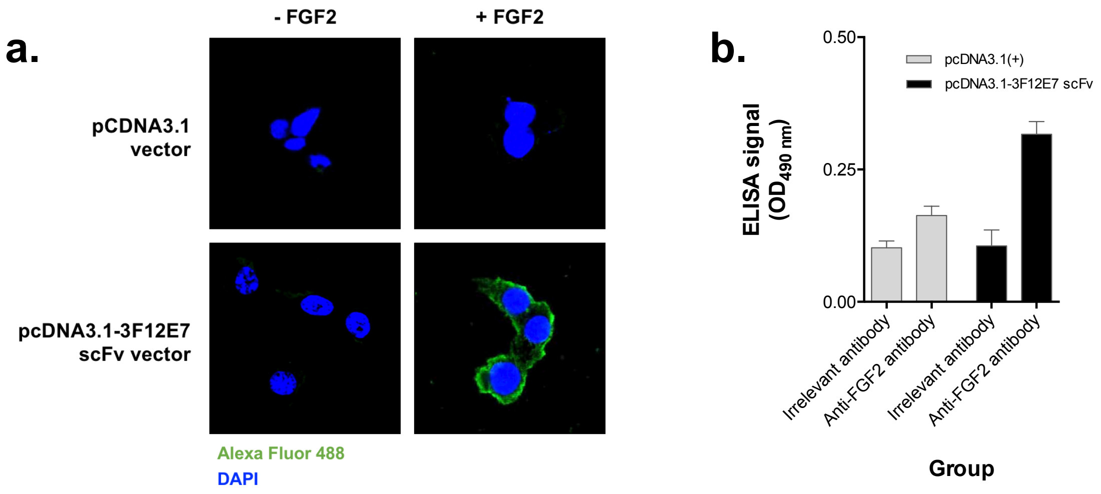

Supplementary Figure S1. Expression of 3F12E7 anti-FGF2 scFv in mammalian cells. (a) Immunofluorescence detection of 3F12E7 scFv in the surface of HEK293 cells expressing this mAb, as described in **Supplementary Methods**. DAPI stains nuclei. Image acquired using a 63x objective. **(b)** Cell-bound ELISA detection of the scFv expression in HEK293 cells transfected with pcDNA3.1-3F12E7 scFv vector. The 3F12E7 scFv was detected indirectly with polyclonal anti-FGF2 antibodies, after cell incubation with recombinant FGF2. Data are mean±s.d. of three independent experiments (n=3).

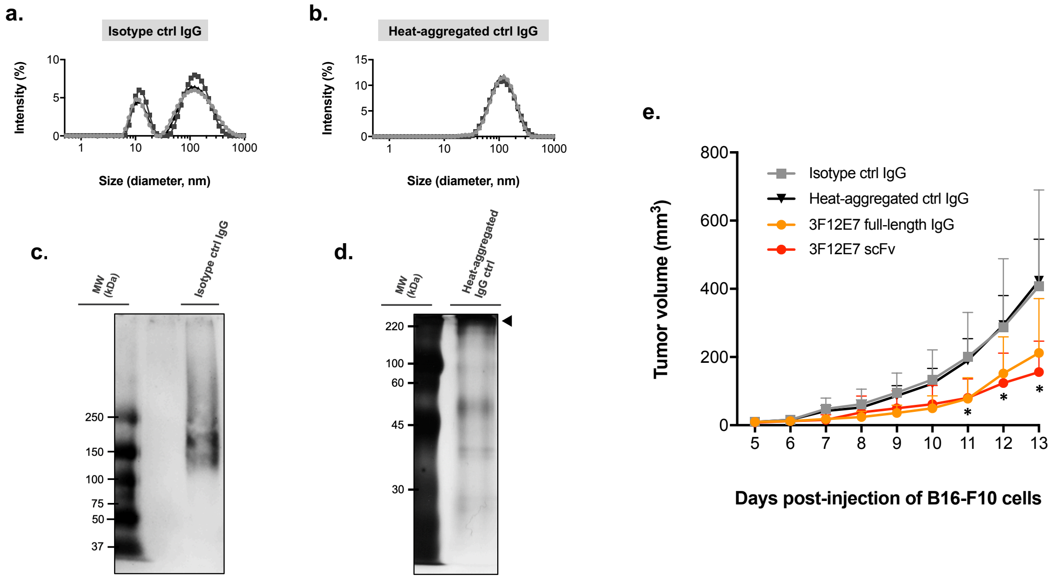

Supplementary Figure S2. Colloidal characterization and anti-tumor effect of soluble heat-aggregated IgG complexes. DLS analysis for **(a)** isotype ctrl IgG and **(b)** heat-aggregated IgG complexes. Mean particle size results for each mAb are expressed by signal intensity. Each line denotes data obtained for three independent samples. BN-PAGE profile for **(c)** isotype ctrl IgG and **(d)** heat-aggregated IgG complexes. Arrowhead indicates the detection of aggregated IgG complexes. Gels were stained with Coomassie Blue R-250. **(e)** Heat-aggregated IgG complexes did not lead to reduced tumor growth. Treatment started four days after subcutaneous injection of B16-F10 cells. Data on tumor growth curve are mean±s.d. Experimental groups: isotype ctrl IgG, n=5; heat-aggregated ctrl IgG, n=5; 3F12E7 full-length IgG, n=5; 3F12E7 scFv, n=5. *P<0.05 compared with isotype IgG and heat-aggregated IgG ctrl groups; *one-way* ANOVA/Bonferroni's post-test. In all assays, heat-aggregated IgG samples were filtered through a 0.22-μm filter prior to their use.

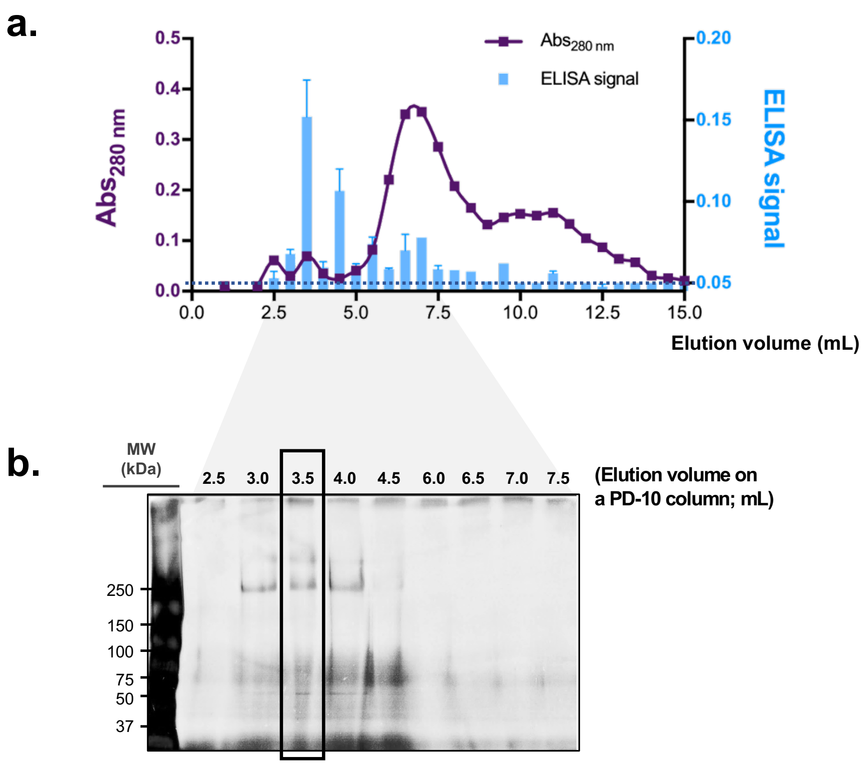

Supplementary Figure S3. Chromatography and BN-PAGE analysis of biotin-labeled 3F12E7 scFv preparation. (a) Size-exclusion chromatography analysis (on a PD-10 column) of 3F12E7 scFv following biotin labeling procedure. Collected elution fractions were evaluated for absorbance at 280 nm. FGF2 binding of each one was assessed by direct ELISA, as described in **Supplementary Methods**. Dashed line indicates ELISA background signal. **(b)** BN-PAGE analysis of the indicated chromatographic fractions. Gel was stained with silver nitrate. Fraction at 3.5 mL (indicated by black rectangle) was used in immunoblotting assay.

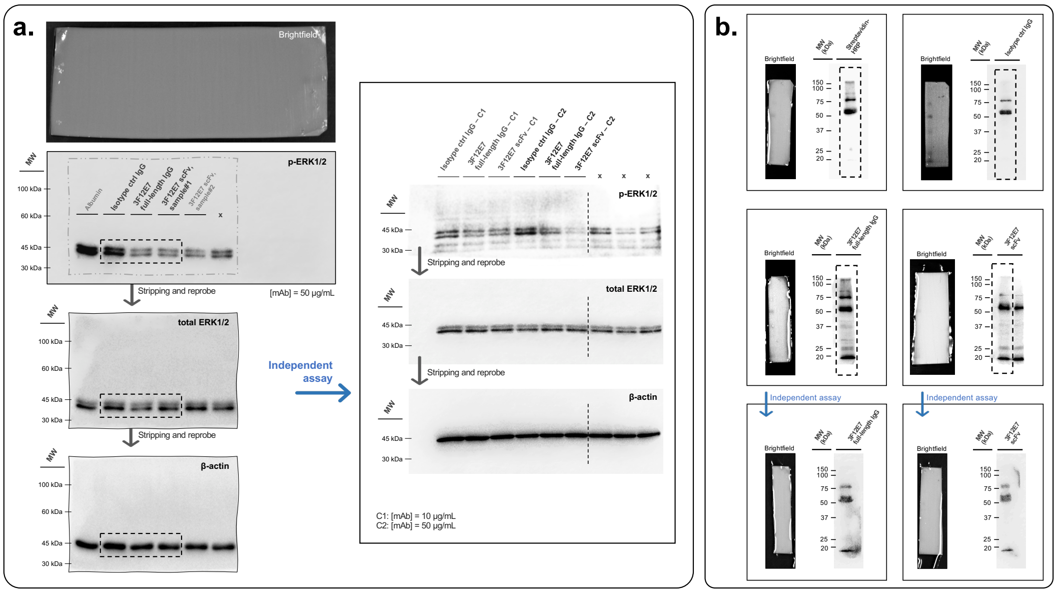

Supplementary Figure S4. Full-length immunoblots from images in (a) Fig. 2d and (b) 3e. The dashed boxes indicate the regions included in the main text. Results of additional independent assays are also provided. MW, molecular weight (kDa).
